# Green One Pot Solvent-Free Synthesis of Pyrano[2,3-*c*]-Pyrazoles and Pyrazolo[1,5-*a*]Pyrimidines 

**DOI:** 10.3390/molecules15096619

**Published:** 2010-09-20

**Authors:** Hamad M. Al-Matar, Khaled D. Khalil, Aisha Y. Adam, Mohamed H. Elnagdi

**Affiliations:** 1 Chemistry Department; Faculty of Science; University of Kuwait; P.O. Box 5969; Safat; 13060 Kuwait; 2 Chemistry Department; Faculty of Science; Cairo University, Giza, 12613, Egypt

**Keywords:** pyranopyrazole, *multi*-component synthesis, aminopyrazolinone, pyrazolo[1,5-*a*]pyrimidine, cyanoacetic acetic anhydride, *NOE* difference experiments

## Abstract

Pyrano[2,3-*c*]pyrazoles are obtained via mixing ethyl acetoacetate, hydrazine hydrate, aldehydes or ketones and malononitrile in the absence of solvent. These same products were also obtained by reacting arylidenemalononitriles **3** with 3-methyl-2-pyrazolin-5-ones. *NOE* difference experiments confirmed that these products exist solely in the 2*H* form. Similar treatments of 3-amino-2-pyrazolin-5-one with arylidene-malononitrile afforded adduct **6**. Similarly mixing ethyl cyanoacetate, hydrazine hydrate, aldehydes, with malononitrile gave the same product **6**. A novel synthesis of 4-oxo-4H-pyrano[2,3-*c*]pyrazole (**8**) could be achieved via reacting 3-methyl-2-pyrazolin-5-one with a mixture of cyanoacetic acid and acetic anhydride. Similar treatment of 3-aminopyrazole **11** with the benzylidene-malononitrile produced the pyrazolo[2,3-*a*]pyrimidines **12a,b**.

## Introduction

The considerable biological activities of 2-amino-4-substituted pyrano[2,3-*c*]pyrazole-3-carbonitriles **1**, have stimulated considerable research directed for synthesis of derivatives of this ring system. These compounds were first obtained by H. Otto in 1974 [1] by adding malononitrile to 4-arylidene-3-methyl-2-pyrazolin-5-one. In 1981 [2] we reported alternate synthesis of this same product, via addition of arylidenemalononitrile to 3-methyl-2-pyrazolin-5-one and assigned the same structure, despite a claim to the contrary in a relatively recent citation of our work [3]. Since then numerous authors [4,5,6,7,8,9,10,11,12] have used this approach for the synthesis of a variety of derivatives of **1**, although several have ignored our original synthesis [11,12]. Despite the fact that it could be established by X-ray that these compounds exist exclusively in the 2*H* form [5,6,7,8,9,10], several subsequent papers ignored this finding [11,13]. Recently we used *NOE* difference experiments to establish that these products also exist in the 2*H* form in DMSO solutions [14]. Recently derivatives of **1** have been obtained via direct one pot synthesis upon mixing ethyl acetoacetate, hydrazine hydrate, aromatic aldehydes and malononitrile [15,16,17,18]. We became interested to see if the multi-component synthesis of **1** can be extended to enable synthesis of other substituted pyranopyrazoles and if the products are obtained this way in yields comparable to those originally obtained. In the same time we have looked into a report in the literature [12] of the isolation of acyclic adducts **5** from reaction of 3-methyl pyrazoline-5-one **2** (R = CH_3_) and arylidene-malononitrile **3**, as isolation of this product stands out as an oddity among plenty of reports indicating that derivatives of **1** are the only isolable products in these reactions [1,2,3]. We have also investigated the possible replacement of ethyl acetoacetate with ethyl cyanoacetate and 3-oxoalkanenitriles in the reported multi-component reaction. This work enabled us to introduce an efficient approach for the synthesis of pyranopyrazoles that could be shown via *NOE* difference experiments to exist in the 2*H* form. Moreover new syntheses of 1-substituted aminopyrazoline-5-ones and pyrazole[1,5-*a*]pyrimidines could be achieved. The one step synthesis approach seems to offer an attractive green methodology that saves both solvent and time compared to the multi-step approach.

## Result and Discussion

**Scheme 1:**
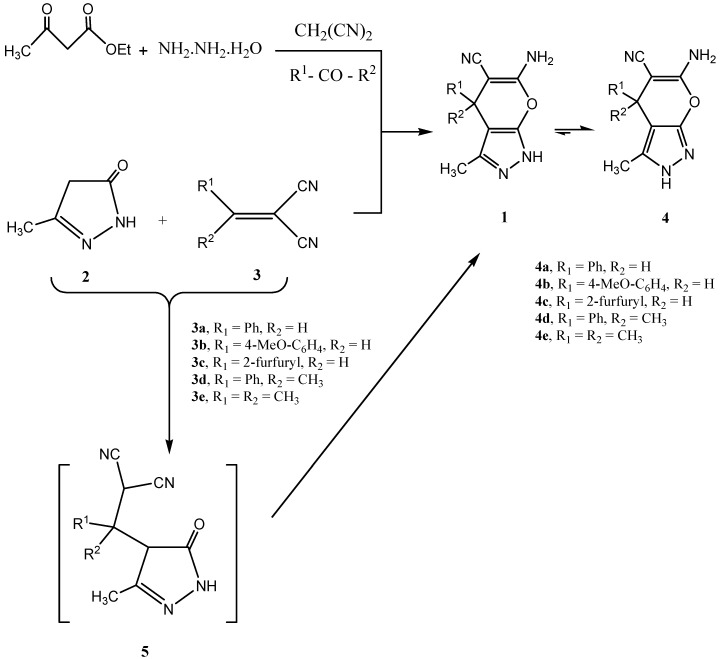
Proposed mechanism for the formation of 2-H-pyranopyrazoles **4a-e**.

Mixing equimolecular amounts of ethyl acetoacetate with hydrazine hydrate, benzaldehyde and malononitrile has produced **1a** or **4a**. This same product could be obtained in almost the same yield by reacting **2** and **3** in ethanol in the presence of chitosan or, as originally reported, in the presence of piperidine. Despite the recently claimed isolation of Michael adduct **5**, this could not be repeated even when the reaction was conducted at room temperature for a short period. Only either unchanged starting materials or cyclic products were isolated. It is of value to report that after an induction period the reaction is exothermic and temperature control is somewhat difficult. Similarly when benzaldehyde was replaced with anisaldehyde or furfural in the above multi-component reaction pyranopyrazoles **4b** and **4c** were produced (Scheme 1). All these proved to exist, in contrast to literature reports for **4b** and **4c**, in the 2*H* form **4**. These same pyranopyrazoles were also produced in the classical route via reaction of **2** and **3** (R_1 _= H) in the presence of chitosan or piperidine. ^1^H-NMR and ^13^C-NMR indicated the presence of one tautomer **4** (the 2*H* form). In addition, *NOE* difference experiments could readily confirm the predominance of form **4** as irradiating the CH_3_ signal at σ 1.9 ppm resulted in an enhanced NH signal at σ 12.17 ppm, in addition to aryl proton enhancement. Thus we conclude that the 2-*H* form is the predominant one and this is also supported by the previous X-ray investigations [5,6,7,8,9,10], although a fast tautomerization with the 1*H* form can't be excluded. We have found that a mixture of ethyl acetoacetate, hydrazine hydrate, malononitrile and acetophenone or acetone also affords pyranopyrazoles **4d,e** also proven to exist in the 2*H* form as major product, as indicated (cf. Scheme 1). 

We have then looked into possible replacing ethyl acetoacetate by ethyl cyanoacetate hoping to obtain aminopyranopyrazoles, pyrazolopyrimidines or pyrazolopyridines. Although the goals were not met, the obtained results seem of value. A mixture of ethyl cyanoacetate, carbonyl compound, hydrazine hydrate and malononitrile was reacted to yield a product that could also be isolated from the reaction of 3-amino-2-pyrazoline-5-one with **3**. Several structures seemed possible. Structure **7** could however be assigned to the reaction product based on ^1^H-NMR that revealed a CH_2_ singlet at σ 2.50 ppm which proved through *HMBC* to be linked to pyrazoline carbon at σ 41.39 ppm. It is thus assumed that initially an aminopyrazolone is formed that then reacted with an *in-situ* formed **3** to give adduct **7**. In the cases of **7a-d**, the adduct could not be isolated as it was auto-oxidized to **6**. 

Recently we reported that mixtures of cyanoacetic acid and acetic anhydride can effectively cyanoacylate electron rich aromatics in the absence or in presence of a catalyst [19]. Reaction of this mixture with **2** afforded also a cyanoacylated product. IR and ^13^C-NMR indicated involvement of cyano function in this product in a subsequent cyclization. Several structures seemed possible for this product (cf. structures **8, 10** in Scheme 3). ^1^H-NMR and ^13^C-NMR indicated the presence of only one tautomer **8** (in 2*H* form). Structure **8** could also be readily established based on *NOE* difference experiments as irradiating the Me protons at σ 2.26 ppm enhanced only the NH at σ 5.9 ppm while the NH_2_ protons were not affected, which excludes both structures **9** and **10**, thus pointing out that the 2*H* form is the predominant one; this is also supported by the previous X-ray investigations [5,6,7,8,9,10]. However again the rapid tautomerization to the 1*H* form **9** can't be excluded. 

**Scheme 2:**
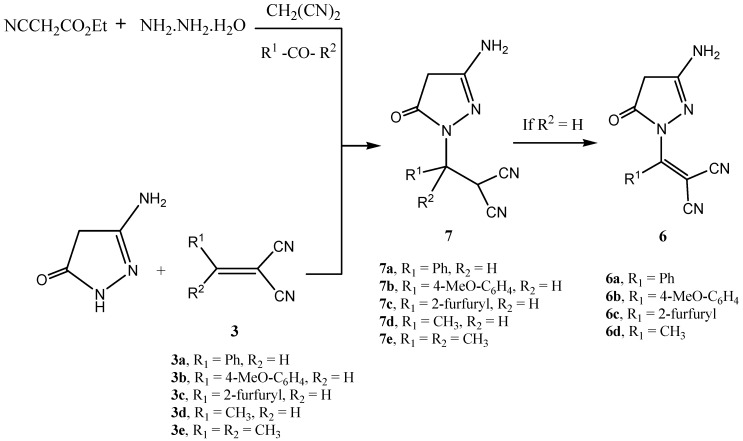
Multi-component reaction of aminopyrazolone with arylidene-malononitrile.

**Scheme 3:**
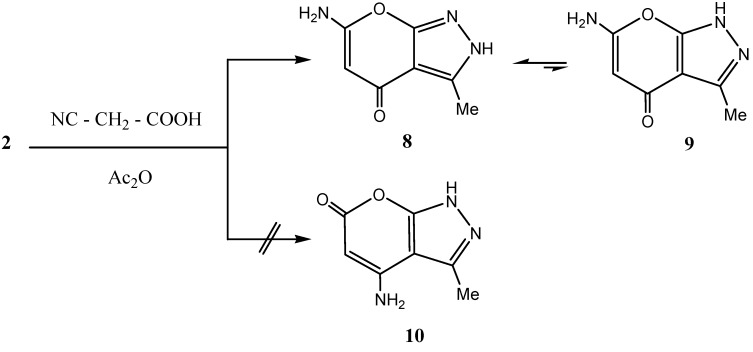
Synthesis of 6-Amino-3-methyl-2H-pyrano[2,3-c]pyrazol-4-one **8**.

**Scheme 4:**
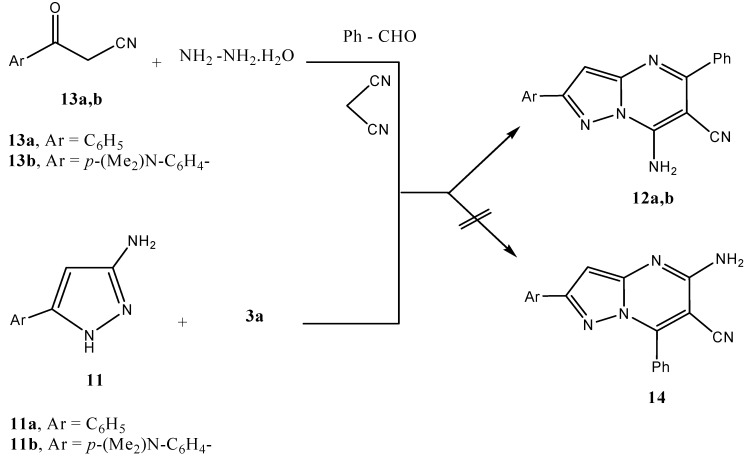
Multi-component reaction of aminopyrazole **11** with benzylidene-malononitrile **3a**.

We have previously reported that **11a** reacts with **3a** to yield **12a** [20]. We now report that **12b** could be directly formed via a mixture of **13b**, hydrazine hydrate, benzaldehyde, and malononitrile. Although the two reactions may also yield **14**, the 7-amino structure is established based on ^15^N *HMBC* where the NH_2_ protons showed a cross peak with *sp^3^* hybridized N-atom. This methodology has been extensively utilized recently to establish structures of the reaction products of α,β-unsaturated nitriles with aminopyrazoles (Scheme 4).

## Experimental

### General

Melting points were recorded on a Gallenkamp apparatus and are uncorrected. Infrared spectra (KBr) were determined on a Perkin-Elmer 2000 FT-IR system. NMR measurements were determined on a Bruker DPX spectrometer, at 600 MHz for ^1^H-NMR and 125 MHz for ^13^C-NMR, in DMSO-*d*_6_ as solvent and using TMS as internal standard. Mass spectra were measured on MS 30 and MS 9 (AEI) spectrometers, with EI 70 eV. Elemental analyses were measured by means of LECO CHNS-932 Elemental Analyzer. Copies of original data can be provided upon request. 

### Synthesis of pyranopyrazoles ***4a-e***

#### Method A

A mixture of ethyl acetoacetate (1.30 g, 10 mmol), hydrazine hydrate (1.00 mL, 80%), malononitrile (0.66 g, 10 mmol), carbonyl compound (benzaldehyde, *p*-methoxybenzaldehyde, furfural, acetophenone or acetone, 10 mmol), was mixed in presence of 5 drops of triethylamine. The mixture was refluxed for 6 h. The crude product was cooled and collected by filtration. Then, the product was recrystallized from ethanol to give the corresponding pyranopyrazoles **4a-e**.

#### Method B

A mixture of pyrazolone (0.98 g, 10 mmol) and a carbonyl compound (benzaldehyde, *p*-methoxybenzaldehyde, furfural, acetophenone or acetone) arylidene (10.0 mmol) was mixed with 5 drops of triethylamine. The mixture was refluxed for 6 h. The crude product was purified as mentioned in method A.

*6-Amino-3-methyl-4-phenyl-2,4-dihydropyrano[2,3-c]pyrazole-5-carbonitrile* (**4a**). White powder (m.p. 245 °C, literature [2] m.p. 245–246 °C), (2.06 g, % yield = 82); IR (KBr): *υ* = 3372 (groups of NH, NH_2_), 2190.74 (CN) cm^-1^; ^1^H-NMR: *δ* = 1.78 (s, 3H, CH_3_), 5.18 (s, 1H, C-4 pyran), 6.89 (s, 2H, NH_2_), 7.16–7.33 (m, 5H, phenyl), 12.11 (s, 1H, NH); ^13^C-NMR: *δ* = 9.8 (CH_3_), 36.2 (pyran C), 57.2 (C-CN), 120.8 (CN), 126.8, 127.5, 128.4, 135.6, 144.5 (aromatic carbons); MS, *m/z* (%), 252.1 (23),175.1 (M^+^, 100); Anal. Calcd. for C_14_H_12_N_4_O: C, 66.65; H, 4.79; N, 22.21. Found: C, 66.50; H, 4.65; N, 22.05. 

*6-Amino-4-(4-methoxyphenyl)-3-methyl-2,4-dihydropyrano[2,3-c]pyrazole-5-carbonitrile* (**4b**). White powder (m.p. 210 °C, literature [2] m.p. 212 °C), (2.50 g, % yield = 88.8); IR (KBr):*υ* = 3483.78 (NH_2_), 3255.25 (NH), 2191.7 (CN) cm^-1^; ^1^H-NMR: *δ* = 1.79 (s, 3H, CH_3_), 3.73 (s, 3H, O-CH_3_), 4.54 (s, 1H, C-4 pyran), 6.83 (d, 2H, *J* = 8.2 *Hz*, p-anisyl-H), 6.88 (d, 2H, *J* = 8.2 *Hz*, p-anisyl-H), 7.07 (s, 2H, NH_2_), 12.08 (s, 1H, NH); ^13^C-NMR: *δ* = 9.8 (CH_3_), 35.4 (pyran C), 54.9 (CH_3_-O), 57.6 (ethylene C), 117.8 (CN), 105.64, 110.24, 113.74, 136.48, 142.28, 154.74, 155.72 (furan and pyrazole carbons) 160.67 (ethlyene C); MS, *m/z* (%), 175.1 (M^+^, 100), 282.1 (40); Anal. Calcd. for C_15_H_14_N_4_O_2_: C, 63.82; H, 5.10; N, 19.85. Found: C, 63.16; H, 4.89; N, 19.74. 

*6-Amino-4-(furan-2-yl)-3-methyl-2,4-dihydropyrano[2,3-c]pyrazole-5-carbonitrile* (**4c**). White powder (m.p. 219 °C, literature [21] m.p. 218 °C), (2.08 g, % yield = 86); IR (KBr): *υ* = 3357.5 (NH_2_), 3315.0 (NH), 2187.8 (CN) cm^-1^; ^1^H-NMR: *δ* = 1.98 (s, 3H, CH_3_), 4.78 (s, 1H, C-4 pyran), 5.96 (s, 2H, NH_2_), 6.18 (d, 1H, *J* = 1.6 Hz, C-3 furan), 6.37 (dd, 1H, *J* = 1.6, *J* = 3.2 Hz, C-4 furan), 7.53 (d, 1H, *J* = 3.2 Hz, C-5 furan), 12.16 (s, 1H, NH); ^13^C-NMR: *δ* = 9.58 (CH_3_), 29.81 (C-4, pyran), 53.96 (C-3 pyran), 95.11 (C-3 furan), 105.64 (C-4 furan), 110.24 (C-4 pyrazoles), 120.60 (CN), 135.83 (C-3 pyrazole), 142.28 (C-5 furan), 154.81 (C-5 pyrazole), 155.72 (C-2 furan), 161.48 (C-2 pyran); MS, *m/z* (%), 242 (M^+^, 100), 175.1 (86); Anal. Calcd. for C_12_H_10_N_4_O_2_: C,59.50; H, 4.16; N, 23.13. Found: C, 59.39; H, 4.02; N, 22.97. 

*2-[1-(3-Methyl-5-oxo-4,5-dihydro-1H-pyrazol-4-yl)-1-phenyl-ethyl]-malononitrile* (**4d**). Pale yellow crystals (m.p. 200 °C, literature [22] 200 °C), (1.40 g, % yield = 53); IR (KBr): *υ* = 3380, 3320, 3190 (NH and NH_2_), 2189 (CN) cm^-1^; ^1^H-NMR: *δ* = 1.76 (s, 6H, two CH_3_), 6.78 (s, 2H, NH_2_), 7.17–7.33 (m, 5H, phenyl), 12.12 (s, 1H, NH); ^13^C-NMR: *δ* = 10.1 (CH_3_), 27.2 (CH_3_), 37.4 (C-4 pyran), 64.5 (C-3 pyran), 102.5 (C-5 pyran), 118.9 (CN), 126.1, 126.4, 128.0 (aromatic carbons), 134.9 (C-6 pyran), 147.3 (C-1 phenyl), 153.9 (C-5 pyrazole), 159.9 (C-2 pyran); MS, *m/z* (%), 266.1 (M^+^, 7), 251.1 (100); Anal. Calcd. for C_15_H_14_N_4_O: C, 67.65; H, 5.30; N, 21.04. Found: C, 67.46; H, 5.24; N, 20.81. 

*6-Amino-3,4,4-trimethyl-2,4-dihydropyrano[2,3-c]pyrazole-5-carbonitrile* (**4e**). Pale yellow powder (m.p. 175 °C), (1.86 g, % yield = 91); IR (KBr):*υ* = 3472.2 (NH), 3240 (NH_2_), 2185.9 (CN) cm^-1^; ^1^H- NMR: *δ* = 1.17 (s, 6H, two CH_3_), 2.24 (s, 3H, CH_3_), 6.65 (s, 2H, NH_2_), 12.05 (s, 1H, NH); ^13^C-NMR: *δ* = 11.27 (CH_3_), 30.60 (two CH_3_, pyran), 63.96 (C-4 pyran), 102.67 (C-3 pyran), 120.41 (C-4 pyrazole), 135.30 (CN), 135.35 (C-3 pyrazole), 154.15 (C-5 pyrazole), 160.41 (C-2 pyran); MS, *m/z* (%), 204.1 (M^+^, 7), 189.1 (100); Anal. Calcd. for C_10_H_12_N_4_O: C, 58.81; H, 5.92; N, 27.43. Found: C, 58.76; H, 5.86; N, 27.39. 

### Reaction of 3-amino-5-pyrazolone with arylidene-malononitriles ***3***

#### Method A

A mixture of ethyl cyanoacetate (1.13 g, 10 mmol), hydrazine hydrate (1.00 mL, 80%), malononitrile (0.66 g, 10.0 mmol) and carbonyl compound (benzaldehyde, *p*-methoxybenzaldehyde, furfural, acetaldehyde or acetone, 10.0 mmol) was refluxed in presence of 5 drops of triethylamine. The crude product was cooled and collected by filtration. Then, recrystallized from ethanol to give the pyrazolopyrimidine and acyclic addition products **6a-d** and **7e**, respectively.

#### Method B

A mixture of 3-amino-5-pyrazolone (0.99 g, 10 mmol) and a carbonyl compound (benzaldehyde, *p*-methoxybenzaldehyde, furfural, acetaldehyde or acetone) arylidene (10.0 mmol) was refluxed for 3 h in presence of 5 drops of triethylamine. The crude product was purified as in method A.

*7-Amino-2-oxo-5-phenyl-1,2-dihydropyrazolo[1,5-a]pyrimidine-6-carbonitrile* (**6a**). Pale yellow crystals (m.p. 288 °C), (1.4 g, % yield = 56); IR (KBr): *υ* = 3244.7 (br. bands, NH, NH_2_), 2217.7 (CN), 1599.7 (CO) cm^-1^; ^1^H-NMR: *δ* = 2.50 (s, 2H, pyrazole CH_2_), 7.48–7.57 (m, 5H, phenyl), 8.49 (s, 2H, NH_2_); ^13^C-NMR: *δ* = 41.4 (C-4 pyrazolone), 74.3 (C-CN), 116.3 (CN), 115.5, 127.9, 128.6, 130.2, 134.6, 156.7 (aromatic carbons), 159.2 (C-NH_2_), 159.6 (C=O); MS, *m/z* (%), 251.1 (M^+^, 100); Anal. Calcd. For C_13_H_9_N_5_O: C, 62.15; H, 3.61; N, 27.87. Found: C, 61.80; H, 3.52; N, 27.68. 

*2-[(3-Amino-5-oxo-4,5-dihydropyrazol-1-yl)-(4-methoxyphenyl)-methylene]-malononitrile* (**6b**). White powder (m.p. 314 °C), (1.65 g, % yield = 59); IR (KBr): *υ* = 3396.0, 3319.8 (NH_2_), 2215.8 (two CN), 1640 (C=O) cm^-1^; ^1^H-NMR: *δ* = 3.85 (s, 3H, CH_3_), 5.65 (s, 2H, CH_2_), 7.09 (d, 2H, *J* = 8 Hz, *p*-anisyl H), 7.47 (d, 2H, *J* = 8 Hz, *p*-anisyl H), 8.44 (s, 2H, NH_2_); ^13^C-NMR: *δ* = 55.3 (CH_2_, pyrazolone), 74.2 (C(CN)_2_), 86.2 (CH_3_), 116.9 and 117.6 (Two CN), 113.9, 126.4, 129.8, 156.6, 159.2 (aromatic carbons), 159.3 (C=O), 160.7 (C=C, aliphatic), 153.39 (C-3 pyrazole), 162.35 (C-5 pyrazoles); MS, *m/z* (%), 281.1 (M^+^, 100); Anal. Calcd. For C_14_H_11_N_5_O_2_: C, 59.78; H, 3.94; N, 24.90; Found: C, 59.74; H, 3.89; N, 24.49. 

*2-[(3-Amino-5-oxo-4,5-dihydropyrazol-1-yl)-furan-2-yl-methylene]-malononitrile* (**6c**). White powder (m.p. 320 °C), (1.28 g, % yield = 53); IR (KBr): *υ* = 3279.36 (br. Bands, NH, NH_2_), 2210 (CN), 1601.6 (CO) cm^-1^; ^1^H NMR (DMSO-d_6_): *δ* = 5.65 (s, 2H, CH_2_ pyrazolone), 6.82 (d, 1H, *J* = 1.8 Hz, C-3 furan), 7.34 (dd, 1H, *J* = 1.8 Hz, *J* = 3.1 Hz, C-4 furan), 8.08 (d, 1H, *J* = 3.1 Hz, C-5 furan), 8.45 (s, 2H, NH_2_); ^13^C NMR (DMSO-d_6_): *δ* = 72.4 (CH_2_ pyrazolone), 84.5 (C(CN)_2_), 114.5 (C-3 furan), 117.7 (C-4 furan), 118.2 (CN), 118.5 (CN), 146.7 (C-5 furan), 147.1 (C-3 pyrazoles), 148.1 (C-2 furan), 159.1 (C=O), 161.4 (C=C(CN)_2_); MS, *m/z* (%), 241.0 (M^+^, 100), 197.0 (36); Anal. Calcd. For C_11_H_7_N_5_O_2_: C, 54.77; H, 2.93; N, 29.03. Found: C, 54.57; H, 2.84; N, 28.69. 

*2-[1-(3-Amino-5-oxo-4,5-dihydropyrazol-1-yl)-ethylidene]-malononitrile* (**6d**). White powder (m.p. 320 °C), (1.18 g, % yield = 63); IR (KBr):*υ* = 3316 (br. bands, NH, NH_2_), 2221.6 (two CN), 1651.7 (C=O) cm^-1^; ^1^H-NMR: *δ* = 2.35 (s, 3H, CH_3_), 5.55 (s, 2H, CH_2_), 8.36 (s, 2H, NH_2_); ^13^C- NMR: *δ* = 19.9 (CH_3_), 74.5 (CH_2_ pyrazoles), 86.7 (C(CN)_2_, 116.7, 117.1 (two CN), 156.4 (C-3 pyrazole), 158.1 (C-5 pyrazole), 159.1 (C=C(CN)_2_); MS, *m/z* (%), 189.1 (M^+^, 100), 160.1 (33); Anal. Calcd. For C_8_H_7_N_5_O: C, 50.79; H, 3.73; N, 37.02. Found: C, 50.49; H, 3.70; N, 36.72.

*2-[1-(3-Amino-5-oxo-4,5-dihydropyrazol-1-yl)-1-methyl-ethyl]-malononitrile* (**7e**). White powder (m.p. 202 °C), (1.33 g, % yield = 65); IR (KBr): *υ* = 3413.4, 3344.9 (NH_2_), 2252.5, 2174.4 (two CN), 1698.9 (C=O) cm^-1^; ^1^H-NMR: *δ* = 1.11 (s, 3H, CH_3_), 1.27 (s, 3H, CH_3_), 4.58 (s, 1H, CH(CN)_2_), 5.22 (s, 2H, CH_2_), 6.69 (s, 2H, NH_2_); ^13^C-NMR: *δ* = 24.1 (CH_3_), 26.5 (CH_3_), 32.5 (C(CN)_2_), 47.4 (CH_2_ pyrazole), 61.8 (C(CH_3_)_2_), 115.3, 117.5 (two CN), 153.4 (C-3 pyrazole), 162.4 (C-5 pyrazoles); MS, *m/z* (%), 205.1 (M^+^, 24), 190.1 (100); Anal. Calcd. For C_9_H_11_N_5_O: C, 52.67; H, 5.40; N, 34.13. Found: C, 52.48; H, 5.18; N, 33.68. 

### Cyanoacetylation of methylpyrazolone ***2***

A mixture of cyanoacetic acid (0.85 g, 10 mmol) and acetic anhydride (1.20 g, 10 mmol) was heated for 20 min. and then mixed with methylpyrazolone **2** (0.98 g, 10 mmol). The mixture was refluxed for 6 h. The crude product was treated with cold water and the solid product was then collected by filtration. The product was recrystallized from ethanol to give the corresponding pyranopyrazole **8**.

*6-Amino-3-methyl-2H-pyrano[2,3-c]pyrazol-4-one* (**8**). Light brown crystals (m.p. 234 °C), (0.26 g, % yield = 15.5); IR (KBr): *υ* = 3337.2 (groups of NH, NH_2_), 1654.6 (C=O) cm^-1^; ^1^H-NMR: *δ* = 2.26 (s, 3H, CH_3_), 4.58 (s, 1H, pyran H), 5.91 (s, 1H, NH), 8.05 (s, 2H, NH_2_); ^13^C-NMR: *δ* = 14.3 (CH_3_), 65.9 (C=CNH_2_), 88.5, 150.5, 150.9, 150.9 (aromatic carbons), 152.3 (C-NH_2_), 158.1 (C=O); MS, *m/z* (%),165.1 (M^+^, 100), 98 (70); Anal. Calcd. For C_7_H_7_N_3_O_2_: C, 50.91; H, 4.27; N, 25.44; Found: C, 50.01; H, 4.13; N, 24.93.

### Synthesis of pyrazolopyrimidines ***12a,b***

A mixture of oxoalkanenitriles **13a,b** (10 mmol) hydrazine hydrate (1.00 mL, 80%), benzaldehyde (1.06 g, 10 mmol), and malononitrile (0.66 g, 10 mmol) was refluxed for 4 h. The solid product, so formed, was recrystallized from ethanol.

*7-Amino-2,5-diphenylpyrazolo[1,5-a]pyrimidine-6-carbonitrile* (**12a**). Pale yellow solid (m.p. 253 °C), (% yield = 71); IR (KBr): *υ* = 3350 and 3325 (groups of NH, NH_2_), 2188.3 (CN), cm^-1^; ^1^H-NMR: *δ* = 5.38 (s, 2H, NH_2_), 6.92 (s, 1H, C-4, pyrazole), 7.12–7.68 (m, 10H, two phenyl); ^13^C-NMR: *δ* = 86.4 (C-CN), 92.6 (C-4, pyrazole), 117.6 (CN), 122.1, 124.7, 129.9, 130.5, 132.7, 139.6, 140.0, 142.4, 144.2 (aromatic carbons); MS, *m/z* (%), 311.1 (M^+^, 100), 77 (71); Anal. Calcd. For C_19_H_13_N_5_: C, 73.30; H, 4.21; N, 22.49; Found: C, 73.16; H, 4.13; N, 22.24.

*7-Amino-2-(4-(dimethylamino)phenyl)-5-phenylpyrazolo[1,5-a]pyrimidine-6-carbonitrile* (**12b**). Pale yellow solid (m.p. 270 °C), (% yield = 75); IR (KBr): *υ* = 3340 and 3300 (groups of NH, NH_2_), 2183.0 (CN) cm^-1^; ^1^H-NMR: *δ* = 2.31 (s, 6H, two CH_3_), 5.27 (s, 2H, NH_2_), 7.18 (s, 1H, C-4, pyrazole), 7.21 (d, 2H, *J* = 8.0 H*z*, p-aryl ring), 7.64 (m, 5H, phenyl), 7.84 (d, 2H, *J* = 8.0 H*z*, p-aryl ring); ^13^C-NMR: *δ* = 37.6 (CH_3_), 82.9 (C-CN), 83.1 (C-4, pyrazole), 117.8 (CN), 113.5, 119.2, 121.4, 128.4, 128.8, 131.7, 132.4, 138.3, 139.6, 140.3, 143.6 (aromatic carbons); MS, *m/z* (%), 354.0 (M^+^, 100), 77 (38); Anal. Calcd. For C_21_H_18_N_6_: C, 71.17; H, 5.12; N, 23.71; Found: C, 69.94; H, 5.06; N, 23.31.

## Conclusions

Efficient *multi*-component syntheses of pyrano[2,3-c]pyrazoles and pyrazolo[1,5-*a*] pyrimidines were achieved. The scope of these approaches is promising. The yields in *multi*-component synthetic approaches are almost the same as those utilizing our original synthetic approach but they are greener, avoiding use of solvent, separation and purification steps. Chitosan could be used as a catalyst, thus replacing the homogeneous catalysis of our original syntheses. 

## Supplementary Materials

Supplementary File 1
