# Supplementary figures and images for: Green One Pot Solvent-Free Synthesis of Pyrano[2,3-c]-Pyrazoles and Pyrazolo[1,5-a]Pyrimidines
Source: Molecules. 2010 Sep 20;15(9):6619–29. doi: 10.3390/molecules15096619 (PMC6257727; doi:10.3390/molecules15096619)

## Graphical Abstract

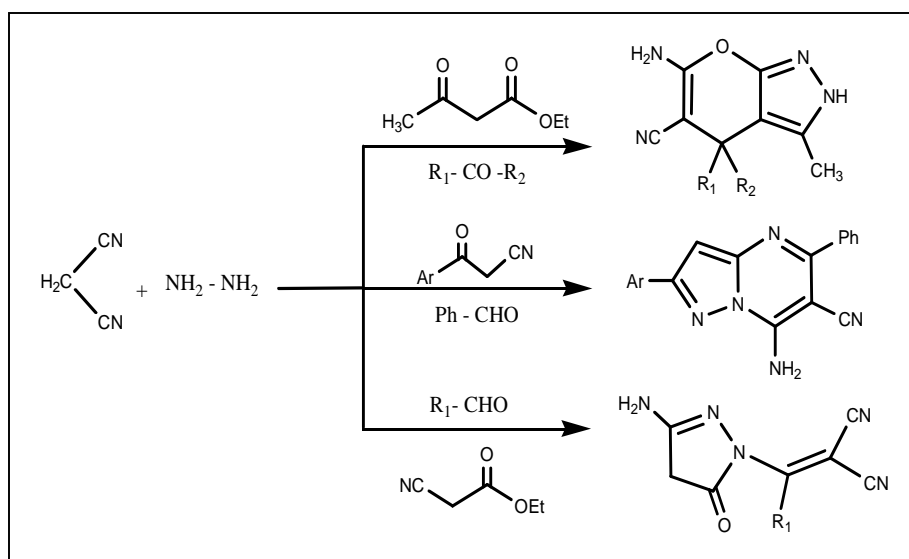

Supplement: Supplementary File 1 [file molecules-15-06619-s001.pdf]
